# Brazilian real-world data of immunotherapy in extensive stage small cell lung cancer

**DOI:** 10.3389/fonc.2025.1425421

**Published:** 2025-02-24

**Authors:** Flavia A. Duarte, Rodrigo Dienstmann, Paulo H. C. Diniz, Matheus C. e Silva, Gilson G. V. Veloso, Georgia S. Arcanjo, Rafael Paes, Tatiane C. Montella, Helena F. C. A. Lima, Bruno L. Ferrari, Eliane V. Macuzo, Carlos G. Ferreira

**Affiliations:** ^1^ Oncoclinicas&Co - Medica Scientia Innovation Research (MEDSIR), Sao Paulo, SP, Brazil; ^2^ Departamento de Clínica Médica, Faculdade de Medicina, Universidade Federal de Minas Gerais, Belo Horizonte, Brazil

**Keywords:** extensive disease, immunotherapy, real-world data, small cell lung cacer, Brazilian data, long-term outcomes

## Abstract

**Introduction:**

The landmarks of Thoracic Oncology in the last two decades have been accompanied by exponential growth in costs, making it imperative to assess the real benefit of incorporating new technologies. Combining immunotherapy (IO) with platinum-etoposide chemotherapy has become the standard of care in first-line treatment of extensive-stage small cell lung cancer (ES-SCLC). However, the absolute difference in overall survival (OS) has reached three months. This study aimed to investigate the impact of this intervention in a real-world cohort.

**Material and methods:**

We retrospectively analyzed data from ES-SCLC patients (pts) from a Brazilian Oncology Group, diagnosed and treated between January 2018 and June 2022. The primary objectives were median OS (mOS) and median time to subsequent treatment (mTST) according to IO exposure in the first-line setting. Secondarily, we intend to compare these results with the literature data and with an internal and contemporary cohort of patients treated with chemotherapy alone.

**Results:**

In total, 85 SCLC patients were included in this analysis. The median follow-up was nine months. First line regimens were atezolizumab + platinum-etoposide in 53%, platinum-etoposide in 36% and platinum-irinotecan in 11%. Among ES-SCLC pts who received IO in their first-line treatment, the mOS was 15.0 months (95% CI: 11.20; 18.80) and the mTST was 8.0 months (95% CI: 6.25; 9.75). As a reference, our internal and contemporary control presented numerically lower mOS and mTST: 9.0 months (95% CI: 2.08; 19.92) and 7.0 months (95% CI: 5.88; 8.12), respectively.

**Discussion:**

Few real-world cohorts are evaluating the impact of IO in ES-SCLC, limited to high-income countries. Our data suggest that IO has a meaningful impact on the outcome of ES-SCLC in daily clinical practice, confirming previous trial results.

## Introduction

Lung cancer represents a significant public health issue and is the leading cause of cancer-related deaths in Brazil and worldwide (1). Meaningful progress has been made regarding its treatment in the past few years, mainly related to precision medicine advances, leading to mortality reduction and overall survival improvement (2). Unfortunately, this progress did not reach all the patients. The histological subtype, known as small cell lung cancer (SCLC), which accounts for approximately 15% of lung cancer cases, is still recognized as a recalcitrant disease (3). Furthermore, this neoplasm is recognized for its dismal prognosis, presenting, characteristically, rapid growth and early metastases, with the diagnosis already at an advanced stage. Moreover, there are no effective screening methods, even in selected populations.

In this scenario, combining platinum and etoposide with immunotherapy, anti-programmed cell death-1 (PD-1), or an anti-programmed cell death ligand-1 (PD-L1) in the first-line setting brought new perspectives to patients diagnosed with this aggressive disease. It emerged as the most remarkable advance over the last 30 years. Many phase 3 studies have demonstrated impacts on OS, especially with prolonged follow-up. In 3 years, for example, the number of patients alive was three times higher in the chemoimmunotherapy group compared to the chemotherapy alone group (4–7). However, despite the unprecedented, the absolute benefit in OS around three months is still considered modest. Concerns have been raised considering the costs associated with this treatment modality, which need to be outweighed.

Therefore, we analyzed data from one of the most prominent Brazilian oncology groups to evaluate the impact of this high-cost therapy in a real-world scenario.

## Methods

### Patient selection

**Figure 1:**
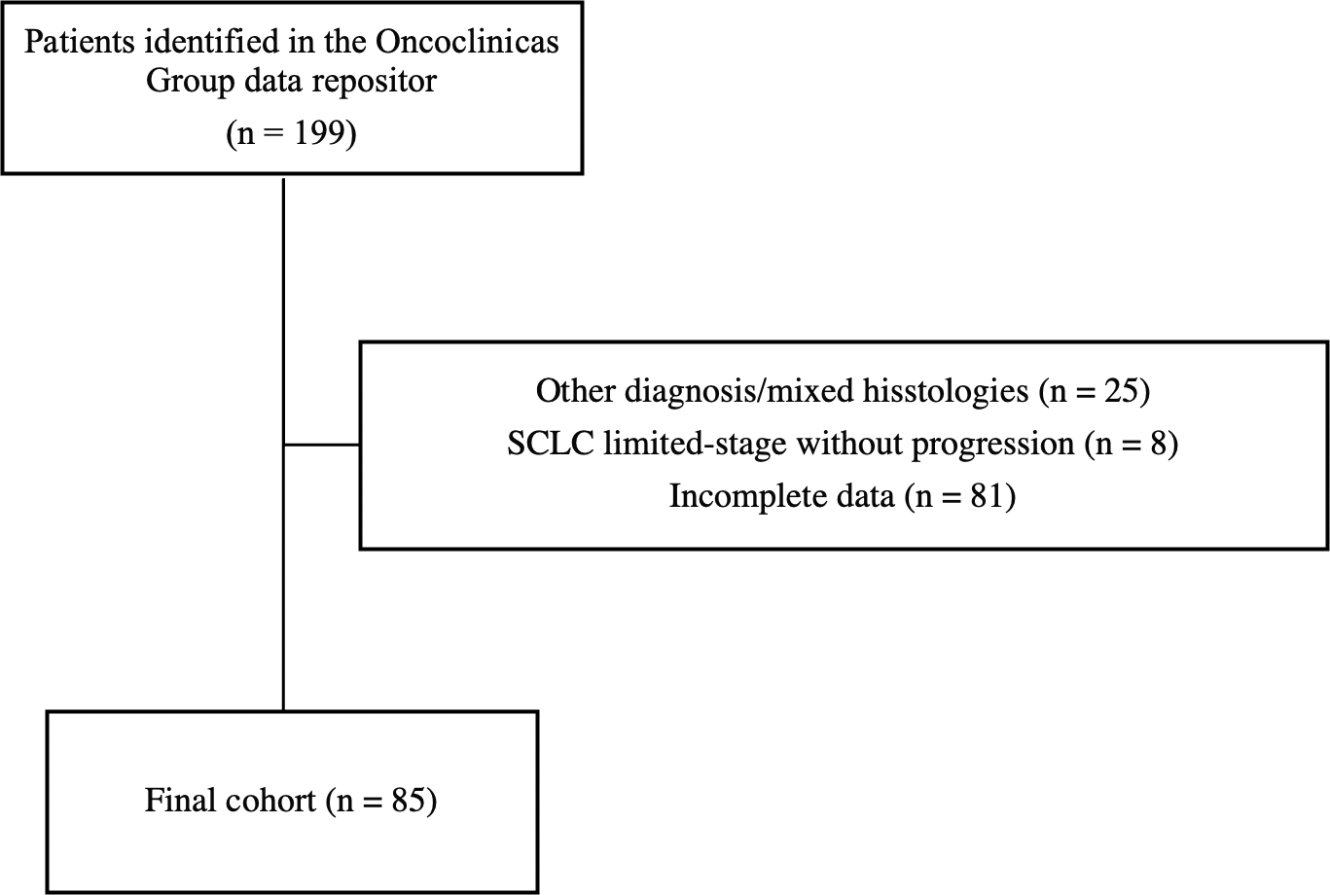
Overall Survival – Immunotherapy + Chemoimmunotherapy versus Chemotherapy (CTx).

We retrospectively analyzed data from patients over 18 years old with histologically confirmed SCLC diagnoses between Jan 1, 2018, and Jun 30, 2022, treated with chemotherapy with or without immunotherapy. Patients diagnosed as extensive stage (ES) or who progressed to this stage during their follow-up were included. On the other hand, patients with mixed histology (associated non-small cell component, large cell neuroendocrine tumors) and those diagnosed with another synchronous tumor were excluded (**Figure 1**).

### Study design

This was a multicenter nationwide retrospective cohort study. We used the repository data of Oncoclinicas Group (OCICdb), a private referral oncologic collaborative group, to identify patients diagnosed with SCLC. This repository combines longitudinal electronic health record (EHR) data from all sites in a cloud‐based platform, which includes structured EHR data (patient demographics, disease stage, anti‐cancer drug prescriptions, and others) with elements from unstructured sources (such as practitioner notes) using technology‐based abstraction techniques. Trained data curators qualify the data using mCODE standards and predefined ontology and actively search for critical outcomes in the patient disease trajectory, including treatment lines, relapse, and death events—external linkage of national death registries to OCICdb guarantees complete information of survival endpoints.

Subsequently, all information on each patient was rechecked through manual assessment of the medical charts. Thus, patients treated in Oncoclinic’s units of the four main Brazilian capitals—Rio de Janeiro, São Paulo, Belo Horizonte, and Salvador—were included in the final evaluation, cities where this resource was available.

### Statistical analysis

The study’s primary endpoints were the median OS and the median time to subsequent treatment (TST) of the patients exposed to IO in the first-line setting. Secondarily, we intent to compare these results with literature data and with an internal and contemporary cohort of patients treated with chemotherapy alone.

OS was calculated from the beginning of first-line treatment for ES to the date of death from any cause. TST was estimated from the start of first-line treatment for ES to the initiation of a new systemic treatment or death. Patients were censored at the last follow-up or data cutoff (Mar 1, 2024). Survival curves were adjusted using the Kaplan-Meier method, and curve comparisons were performed via the log-rank test.

Relevant clinical characteristics that might impact the results were also evaluated. Descriptive statistics were used to summarize the data, which are expressed as numbers and percentages (categorical) and as medians and interquartile range (IQR) (ordinal). For comparison between the categorical characteristics, we used the Chi-square test or the Fisher’s exact test, as appropriate. In contrast, for ordinal variables, when comparing continuous data, we used the Mann-Whitney test or the T-Student test. Statistical significance was assumed at p <0.05 from two-tailed tests. The Cox regression model was performed to determine the characteristics independently associated with OS.

All statistical analyses were performed using SSPS software version 23 (SPSS, Chicago, IL).

## Results

### Patients’ characteristics

In total, 85 SCLC patients were included in this analysis. 53% (45) were treated with immunochemotherapy as their first-line treatment for ES-SCLC and composed the population of interest for the primary objective. Among them, the median age was 69 years, 53% were male, 88% had a smoking history, and only 9% had ECOG performance-status scores of 2 and 3. At diagnosis, 43 of 45 patients presented ES-SCLC. Central nervous system (CNS) metastasis was found in 31%. All the patients were treated with Atezolizumab combined with carboplatin and etoposide as the first line of therapy.

The distribution of clinical characteristics was numerically similar in the internal cohort of patients treated with chemotherapy alone, except for a lower number of patients with CNS metastasis and a higher proportion of patients with ECOG 2 and 3 (**Table 1**). Platinum-etoposide and platinum-irinotecan were the regimens of choice in 77% and 23% of the cases, respectively. In this group, 60% of patients were treated before chemoimmunotherapy approval in Brazil, while 40% were treated after that.

**Table 1:** Clinical characteristics.

Variables	Overall Distribution	Chemotherapy alone 47% (n=40)	Immunotherapy + Chemotherapy 53% (n=45)	p value
Age				
Mean+SD	6910	69 + 11	689	0.84
Gender				
Female	43 (51%)	22 (55%)	21 (47%)	0.517
Male	42 (49%)	18 (45%)	24 (53%)	
ECOG				
0	35 (41%)	17 (43%)	18 (40%)	0.391
1	34 (40%)	13 (32%)	21 (47%)	
2	8 (10%)	4 (10%)	4 (9%)	
3	2 (2%)	2 (5%)	0 (0%)	
Missing information	6 (7%)	4 (10%)	2 (4%)	
Smoker				
Never	6 (7%)	2 (5%)	4 (9%)	0.341
Current/Former	57 (67%)	27 (68%)	30 (67%)	
Missing information	22 (26%)	11 (27%)	11 (24%)	
Extensive disease since initial diagnosis?				
No	5 (6%)	2 (5%)	3 (7%)	1.0
Yes	80 (94%)	38 (95%)	42 (93%)	
CNS Metastasis				
No	65 (76%)	34 (85%)	31 (69%)	0.135
Yes	20 (24%)	6 (15%)	14 (31%)	
Treatment option				
Carboplatin	72 (85%)	27 (68%)	45 (100%)	< 0.001
Cisplatin	13 (15%)	13 (32%)	0 (0%)	

ECOG, Eastern Cooperative Oncology Group;

Chemotherapy alone was considered as the internal and contemporary cohort;

Considering all 85 patients, just 22% (19) had medical chart notes regarding palliative care team follow-up.

### Treatment and survival outcomes

**Figure 2:**
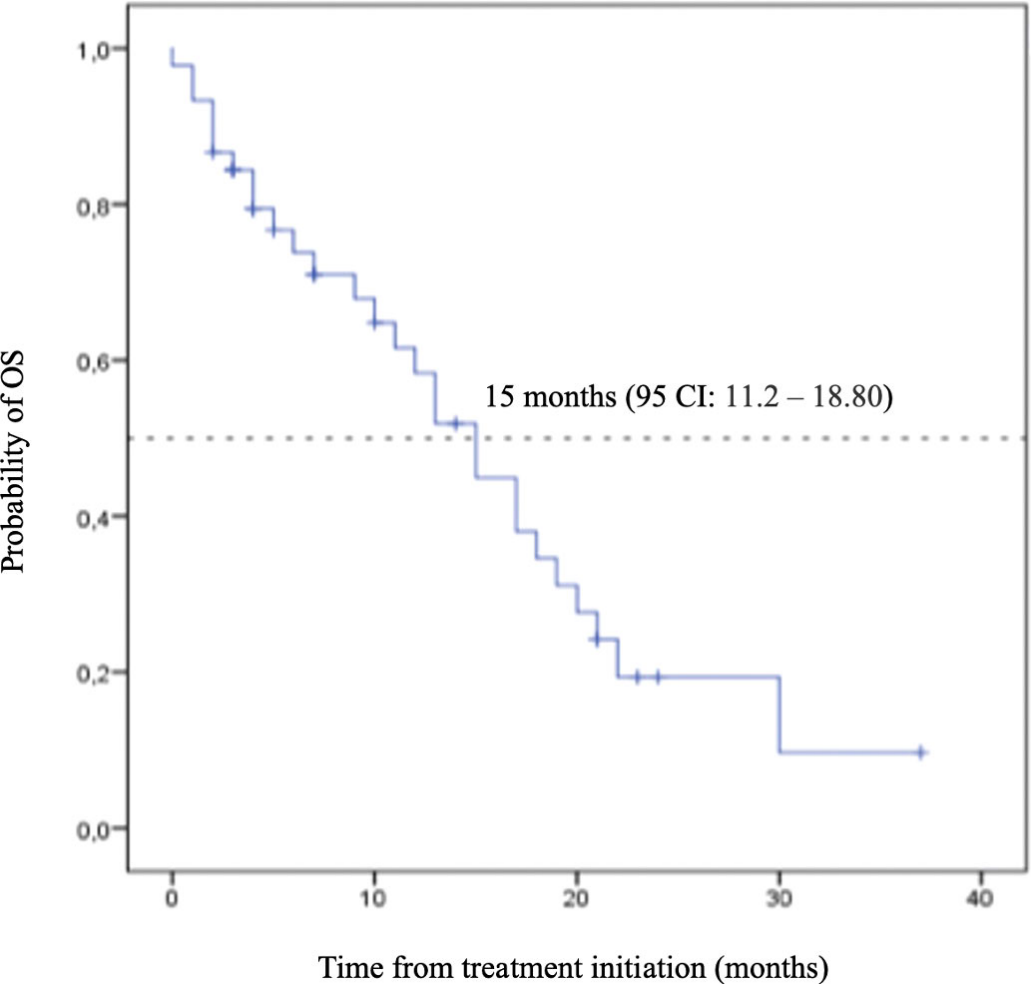
CONSORT diagram. SCLC: small cell lung cancer.

**Figure 3:**
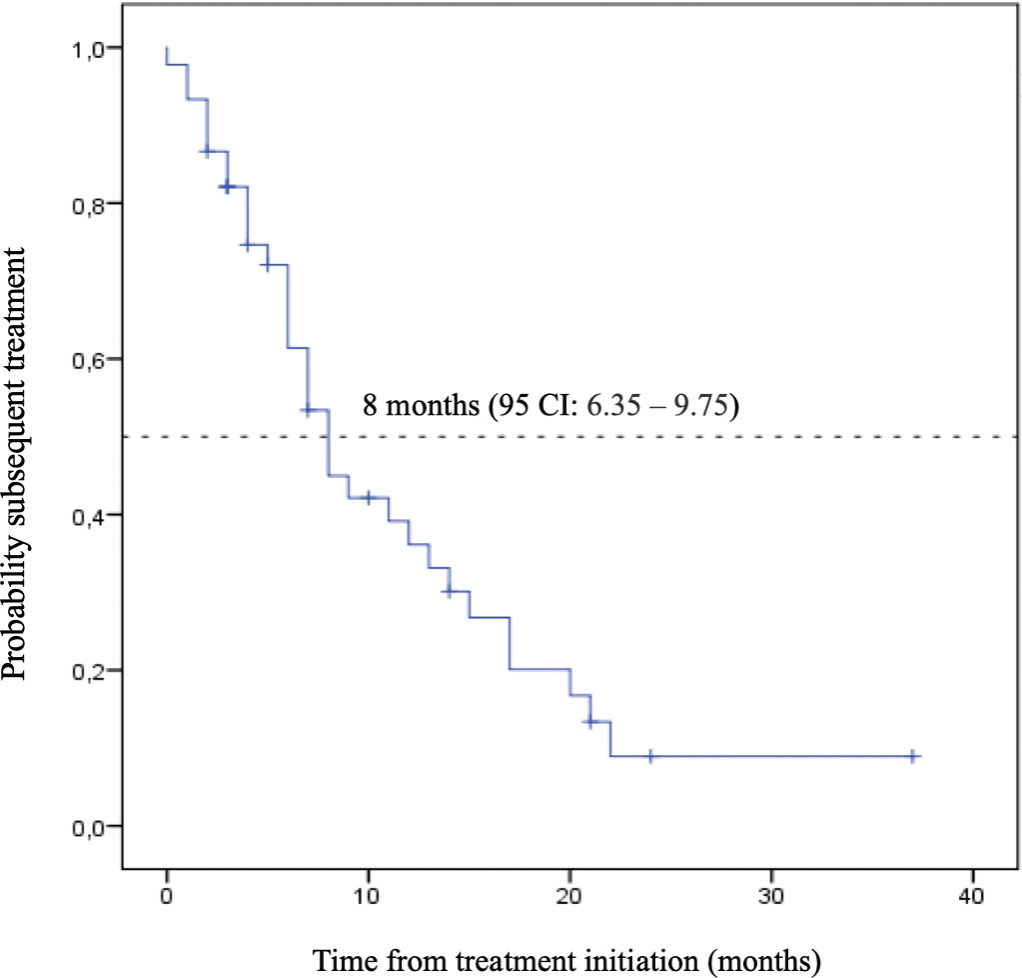
Overall Survival (OS) – Immunotherapy (IO) + Chemotherapy.

With a median follow-up of 9.00 months, the estimated median overall survival of ES-SCLC patients who received IO combined with chemotherapy as their first-line treatment was 15.00 months (95% CI: 11.2 – 18.80). At 18 months, 35% of patients were still alive. Thirty-three percent of the patients received second-line treatment, and the median time for subsequent treatment initiation was 8.00 months (95% CI: 6.35 – 9.75) (**Figures 2**, **3**).

Kaplan-Meier plot of overall survival curve with 95% confidence interval for patients treated with immunotherapy and chemotherapy as the first-line treatment.

Kaplan-Meier plot of time to subsequent treatment curve with 95% confidence interval for patients treated with immunotherapy and chemotherapy as the first-line treatment.

**Figure 4:**
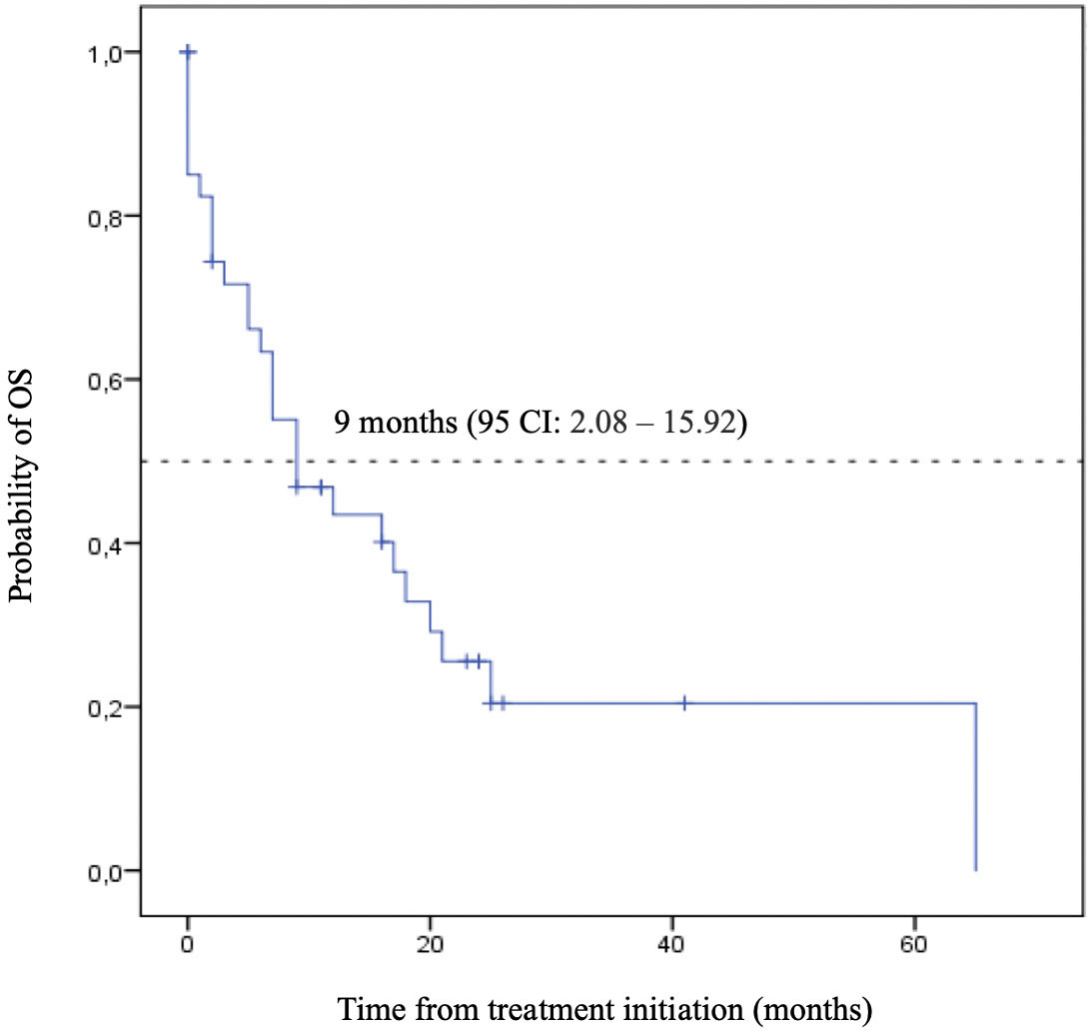
Time to Subsequent Treatment (TST) - Immunotherapy (IO) + Chemotherapy.

**Figure 5:**
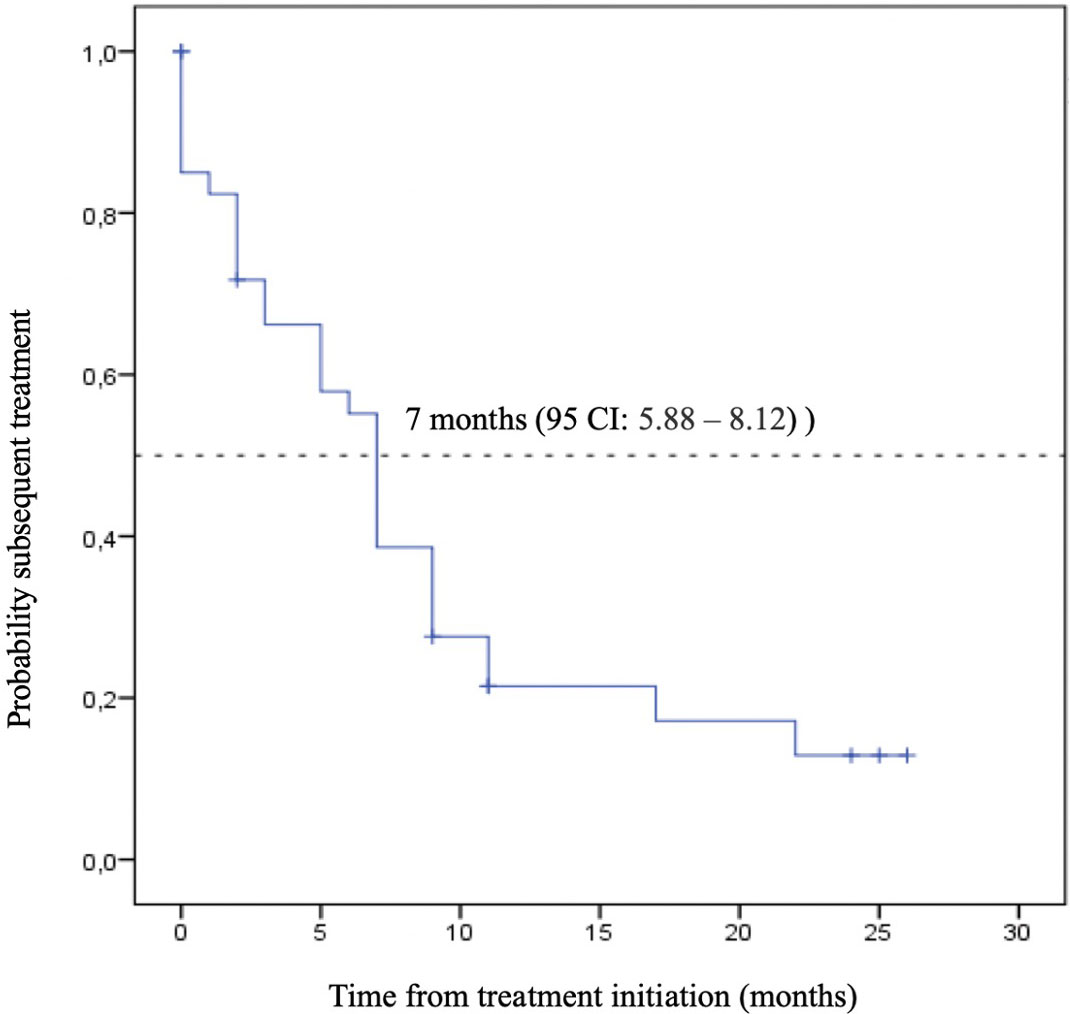
Overall Survival (OS) – Chemotherapy.

As a reference, our internal and contemporary control of chemotherapy-only presented numerically lower mOS and mTST: 9.0 months (95% CI: 2.08 – 15.92) and 7.0 months (95% CI: 5.88 – 8.12), respectively (**Figures 4**, **5**). Second-line treatment was offered to 25% (10) of the patients. The median follow-up for this group was also 9.0 months.

Kaplan-Meier plot of overall survival curve with 95% confidence interval for patients treated with chemotherapy alone as the first-line treatment.

Kaplan-Meier plot of time to subsequent treatment curve with 95% confidence interval for patients treated with chemotherapy alone as the first-line treatment.

**Figure 6:**
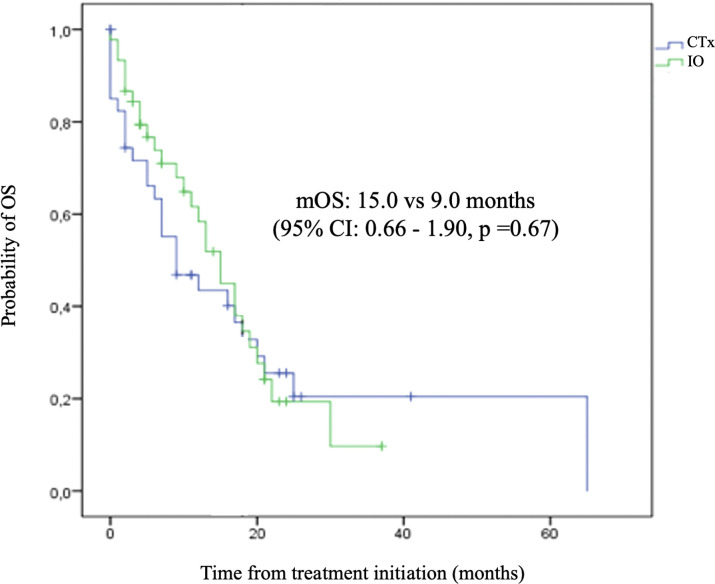
Time to Subsequent Treatment (TST) – Chemotherapy.

Despite the numerical difference observed, the direct comparison among the two cohorts did not show a statistically significant result (**Figure 6**).

Kaplan-Meier plot comparing the overall survival curves with 95% confidence interval for patients treated immunotherapy and chemotherapy versus chemotherapy alone as the first-line treatment.

As expected from original trials, the median number of first-line treatment cycles was superior for the chemoimmunotherapy cohort compared to the chemotherapy alone group 7 versus 4 (p<0.05).

The univariate analyses showed that none of the patients` characteristics were independently associated with overall survival.

## Discussion

The present study, conducted in a real-world scenario, reaffirmed the meaningful benefit of combining immunotherapy with cytotoxic conventional chemotherapy in ES-SCLC patients and was the first to evaluate this combination in the Brazilian population. The mOS was 15.0 months, and mTST was 9.0 months. Interestingly, this clinical benefit was seen even in a population with a higher rate of CNS metastasis compared to the clinical trials and including a few patients with ECOG 2. None of the clinical parameters was independently associated with relevant clinical outcomes, suggesting that the benefit was attributed to the population itself.

SCLC is a challenging and extremely aggressive disease, in which the majority of patients are diagnosed at an advanced stage, and many without clinical performance for oncological treatment (8). Unlike NSCLC, there are currently no biomarkers that are prognostic or predictive of therapeutic response in SCLC. For example, the tumor mutation burden (TMB) is characteristically high in SCLC, although it could not predict IO benefit (9). In this histology, an elevated TMB does not represent many immunogenic neoantigens. Furthermore, the attempt to divide the SCLC into different subtypes either by genetic expression or immunohistochemistry (10) is not a reality in clinical practice, nor has it been prospectively validated.

At present, at least four phase 3 randomized clinical trials have shown statistically significant overall survival improvement with the addition of different anti-PD1 or anti-PD-L1 agents to the standard chemotherapy as the first-line treatment for ES-SCLC: Impower 133, ASTRUM, CAPSTONE, CASPIAN (4–7). The first study to demonstrate this relevant clinical outcome was Impower 133, which evaluated the combination of Atezolizumab, carboplatin, and etoposide, leading to a 24% reduction in the risk of death compared to carboplatin and etoposide alone (mOS of Atezolizumab plus CP/ET: 12.3 months versus mOS of placebo plus CP/ET 10.3 months; hazard ratio, 0.76; 95% CI, 0.60 to 0.95; p = .0154) (11). This result led to Atezolizumab approval in Brazil in 2019. About one year later, another anti-PD-L1, Durvalumab, was approved. Sixty-six percent (n=30) of patients included in our cohort of chemoimmunotherapy initiated their treatment while both drugs were approved in the country, and although there are no objective differences between these two drugs, all the patients were treated with Atezolizumab.

However, immunotherapy costs are still limited, and access to this technology is not universal. Indeed, cost-effectiveness analyses show that, even for developed countries, these therapies may not be cost-effective in this scenario. In a systematic review done by Wang et., the results of most of the sixteen included studies suggested that the combination of immunotherapy with chemotherapy was not cost-effective compared with chemotherapy alone (12).

Therefore, incorporating this expensive strategy into the clinical practice should be judicious. Our analysis showed comparable results to the clinical trials, indicating an appropriate use of chemoimmunotherapy for first-line treatment of ES-SCLC in our population. Even considering its statistical limitations, the comparison with an internal and contemporary cohort also strengthens our finding, as well as reinforces the benefit of immunotherapy in addition to chemotherapy in the real-world scenario with a mOS: 15.0 for the IO group compared to 9.0 months (95% CI: 0.66 - 1.90, p =0.67). Therefore, this data reinforces the need to discuss promoting access to this therapeutic combination in different healthcare settings in Brazil. It provides a basis for healthcare providers and even for the National Commission for the Incorporation of Technologies into the Single Health System evaluations.

Moreover, other aspects highlight the importance of our study. To the best of our knowledge, this is one of the few studies that have evaluated and described a population of SCLC patients in Brazil. The Brazilian population is historically underrepresented in clinical trials. Geographic variations, which reflect genetic and socioeconomic characteristics, are important aspects to consider when applying relevant study conclusions. Secondly, data from middle-income countries are scarce. The main clinical trials included only a few patients from these countries. The IMpower 133, for instance, included only ten patients from South America.

Even considering retrospective data, no published information exists regarding Brazilian or Latin American populations and treatment outcomes (13–15). The Latin American Cooperative Oncology Group (LACOG) has conducted a study to assess the efficacy and safety of Durvalumab associated with platinum-etoposide in previously untreated ES-SCLC in Brazil, and its results will be published in the future (16).

Nevertheless, the retrospective nature of our study is a limitation in itself. Furthermore, the small sample size and underrepresentation of different regions of the country preclude the conduction of analyses adjusted for possible confounding factors. At last, despite being a multicentric cohort, only patients from the Brazilian private healthcare system were included, which represent just 30% of this country’s population (17).

In conclusion, this cohort represents a precocious and mature control of the impact of chemoimmunotherapy strategy impact on first-line treatment of ES-SCLC. Our analyses could be helpful for cost-effective decisions, economic model propositions, and incorporation evaluations in the future. However, further data and prospective studies may be added and are still needed.

## Data Availability

The original contributions presented in the study are included in the article/supplementary material. Further inquiries can be directed to the corresponding author.

## References

[1] Available online at: https://gco.iarc.fr/today/en/dataviz/pie?mode=cancer&group_populations=1&types=1.

[2] Howlader N Forjaz G Mooradian MJ Meza R Kong CY Cronin KA. The effect of advances in lung-cancer treatment on population mortality. N Engl J Med. (2020) 383:640–9. doi: 10.1056/NEJMoa1916623 PMC8577315 32786189

[3] Gazdar AF Bunn PA Minna JD. Small-cell lung cancer: what we know, what we need to know and the path forward [published correction appears in Nat Rev Cancer. 2017 Nov 10. Nat Rev Cancer. (2017) 17:725–37. doi: 10.1038/nrc.2017.87 29077690

[4] Horn L Mansfield AS Szczęsna A Havel L Krzakowski M Hochmair MJ. First-line atezolizumab plus chemotherapy in extensive-stage small-cell lung cancer. N Engl J Med. (2018) 379:2220–9. doi: 10.1056/NEJMoa1809064 30280641

[5] Paz-Ares L Dvorkin M Chen Y Reinmuth N Hotta K Trukhin D. Durvalumab plus platinum-etoposide versus platinum-etoposide in first-line treatment of extensive-stage small-cell lung cancer (CASPIAN): a randomized, controlled, open-label, phase 3 trial. Lancet. (2019) 394:1929–39. doi: 10.1016/S0140-6736(19)32222-6 31590988

[6] Cheng Y Han L Wu L Chen J Shi H Liao H. Effect of first-line serplulimab vs placebo added to chemotherapy on survival in patients with extensive-stage small cell lung cancer: the ASTRUM-005 randomized clinical trial. JAMA. (2022) 328:1223–32. doi: 10.1001/jama.2022.16464 PMC9516323 36166026

[7] Wang J Zhou C Yao W Wang Q Zhong Z Guo Y. Adebrelimab or placebo plus carboplatin and etoposide as first-line treatment for extensive-stage small-cell lung cancer (CAPSTONE-1): a multicentre, randomised, double-blind, placebo-controlled, phase 3 trial. Lancet Oncol. (2022) 23:739–47. doi: 10.1016/S1470-2045(22)00224-8 35576956

[8] Baldotto CS Cronemberger EH de Biasi P de Azevedo CRAS de Lima VCC de Mello MJG. Palliative care in poor-performance status small cell lung cancer patients: is there a mandatory role for chemotherapy? Support Care Cancer. (2012) 20:2721–7. doi: 10.1007/s00520-012-1392-0 22322592

[9] Hellmann MD Callahan MK Awad MM Calvo E Ascierto PA Atmaca A. Tumor Mutational Burden and Efficacy of Nivolumab Monotherapy and in Combination with Ipilimumab in Small-Cell Lung Cancer [published correction appears in Cancer Cell. 2019 Feb 11;35(2):329. Cancer Cell. (2018) 33:853–861.e4. doi: 10.1016/j.ccell.2018.04.001 29731394 PMC6750707

[10] Rudin CM Poirier JT Byers LA Dive C Dowlati A George J. Molecular subtypes of small cell lung cancer: a synthesis of human and mouse model data [published correction appears in Nat Rev Cancer. 2019 Jun 7. Nat Rev Cancer. (2019) 19:289–97. doi: 10.1038/s41568-019-0133-9 PMC6538259 30926931

[11] Liu SV Reck M Mansfield AS Mok T Scherpereel A Reinmuth N. Updated overall survival and PD-L1 subgroup analysis of patients with extensive-stage small-cell lung cancer treated with atezolizumab, carboplatin, and etoposide (IMpower133). J Clin Oncol. (2021) 39:619–30. doi: 10.1200/JCO.20.01055 PMC8078320 33439693

[12] Wang T Li Y Zheng X. Cost-effectiveness of the combination of immunotherapy and chemotherapy for extensive-stage small-cell lung cancer: a systematic review. BMC Health Serv Res. (2023) 23:691. doi: 10.1186/s12913-023-09727-7 37365540 PMC10294391

[13] Elegbede AA Gibson AJ Fung AS Laurie SA Goffin JR Ellis PM. A real-world evaluation of atezolizumab plus platinum-etoposide chemotherapy in patients with extensive-stage SCLC in Canada. JTO Clin Res Rep. (2021) 2:100249. doi: 10.1016/j.jtocrr.2021.100249 34877555 PMC8628038

[14] Nadler ES. Real-world evidence of cancer immunotherapy (CIT) combination treatment in first-line (1L) extensive-stage small cell lung cancer (ES-SCLC). JCO. (2021) 39:8561–1. doi: 10.1200/JCO.2021.39.15_suppl.8561

[15] Sagie S Maixner N Stemmer A Lobachov A Bar J Urban D. Real-world evidence for immunotherapy in the first line setting in small cell lung cancer. Lung Cancer. (2022) 172:136–41. doi: 10.1016/j.lungcan.2022.08.015 36087486

[16] Lopes D Zukin M Calabrich A Cordeiro de Lima V. Assessment of the efficacy and safety of durvalumab associated with platinum-etoposide in previously untreated ES-SCLC in Brazil. JTO. (2023) 18. doi: 10.1016/j.jtho.2023.09.665

[17] Available online at: https://bvsms.saude.gov.br/71-dos-brasileiros-tem-os-servicos-publicos-de-saude-como-referencia/ (Accessed November 16, 2024).

